# Unraveling the selective antibacterial activity and chemical composition of citrus essential oils

**DOI:** 10.1038/s41598-019-54084-3

**Published:** 2019-11-27

**Authors:** Carmen M. S. Ambrosio, Natália Y. Ikeda, Alberto C. Miano, Erick Saldaña, Andrea M. Moreno, Elena Stashenko, Carmen J. Contreras-Castillo, Eduardo M. Da Gloria

**Affiliations:** 1 Department of Agri-Food Industry, Food and Nutrition, “Luiz de Queiroz” College of Agriculture, University of São Paulo, SP, Brazil; 2 Facultad de Ingeniería, Universidad Privada del Norte (UNP), Trujillo, Perú; 3 Facultad de Ingeniería Agroindustrial, Universidad Nacional de Moquegua (UNAM), Moquegua, Perú; 4 School of Veterinary Medicine and Animal Science, University of São Paulo, SP, Brazil; 5 Research Center of Excellence CENIVAM, CIBIMOL, Industrial University of Santander, Bucaramanga, Colombia; 6 Department of Biological Science, “Luiz de Queiroz” College of Agriculture, University of São Paulo, SP, Brazil

**Keywords:** Natural products, Natural products, Antibiotics, Antibiotics

## Abstract

Post-weaning diarrhea (PWD) is an often disease affecting piglets. It is caused mainly by enterotoxigenic *Escherichia coli* (ETEC) colonization in pig gut. Antibiotics has been used to prevent, combat and control PWD and its negative impact on the productivity of pig breeding sector. Nonetheless, antibiotics due to their wide antibacterial spectrum also can reach beneficial gut bacteria, such as *Lactobacillus*. Lately, essential oils (EOs) have emerged as a potential alternative to using antibiotics in animal breeding because of their effect on bacterial growth. Commonly, citrus EOs are by-products of food industry and the availability of these EOs in the worldwide market is huge. Thus, six commercials citrus EOs were evaluated on ETEC strains, as model of pathogenic bacteria, and on *Lactobacillus* species, as models of beneficial bacteria. In overall, citrus EOs exhibited a selective antibacterial activity with higher effect on pathogenic bacteria (ETECs) than beneficial bacteria (*Lactobacillus)*. Brazilian orange terpenes (BOT) oil presented the highest selective performance and caused higher disturbances on the normal growth kinetic of ETEC than on *Lactobacillus rhamnosus*. The action was dose-dependent on the maximal culture density (*A*) and the lag phase duration (λ) of the ETEC. The highest sub-inhibitory concentration (0.925 mg/mL) extended the λ duration to ETEC eight times (14.6 h) and reduced *A* in 55.9%. For *L. rhamnosus*, the λ duration was only extended 1.6 times. Despite the fact that limonene was detected as the major compound, the selective antibacterial activity of the citrus EOs could not be exclusively attributed to limonene since the presence of minor compounds could be implicated in conferring this feature.

## Introduction

The incidence of post-weaning diarrhea (PWD) is a serious problem in the worldwide pig industry, causing severe economic losses due to increased pig morbidity and mortality, decreased animal growth rate and increased need for medication to treat animals^1,2^. PWD is caused mainly by enterotoxigenic *Escherichia coli* (ETEC) that is highly present in the gastrointestinal tract of affected pigs. This pathotype is characterized by production of adhesins, which intermediate bacterial adherence to the intestine^1,3^, and toxins that lead to hypersecretion of water and electrolytes^4,5^. The presence of ETEC in the environment is an important transmission factor since they can survive protected in the manure for about 6 months. Furthermore, *E. coli* multiply rapidly and can reach up to 10^9^ CFU per gram of feces, with the infection produced dependent on the degree of bacterial colonization^6^.

To control PWD outbreaks caused by enterobacteria as ETECs, antibiotics have frequently been included in the diet of weaned piglets as treatment, preventive measure, or growth promoter. However, continuous antibiotic use has been suggested as one cause of the emergence and worldwide dissemination of resistant bacteria. For example, currently, there is great concern about the emergence of plasmid-mediated colistin resistance (*mcr-1*) in *E. coli*, a resistance mechanism to an important antibiotic class in human health, polymyxins, which was largely used in pig production as growth promoter^7–9^, but banned its use for this purpose by the European Union legislation^10^ since 2006, and recently, by China^11^, Japan^12^ and Brazil legislations^13^. However, therapeutic use of colistin is still allowed following the current recommendation of the European Medicine Agency, which restrict the rational use of colistin to treat clinical cases in livestock animals^14^. Moreover, the wide spectrum activity of some antibiotics can affect gut microbiota since they can kill or inhibit both pathogenic and beneficial bacteria. Thus, longtime antibiotic use can provoke decreased microbiota diversity and increased chances of pathogens colonizing the gut^2^. On the other hand, it is well known that *Lactobacillus* is the major group of beneficial bacteria presented in pig gut microbiota, which has been identified as an important group of bacteria able to prevent gut diseases^15^. Possible *Lactobacillus* mechanisms to fight post-weaning infections in piglets have been described as (i) the direct inhibition of pathogen growth and its virulence by secretion of antimicrobial metabolites as bacteriocins, (ii) the modulation of microbiota composition and its activity and, (iii) the stimulation of the host immune system and improvement of intestinal barrier integrity^2^. In this scenario, finding an antimicrobial feed additive with a selective antibacterial activity, high spectrum activity on pathogenic bacteria and a reduced or not effect on beneficial bacteria like *Lactobacillus* would be very desirable. In the last decade, phytogenic compounds like essential oils (EOs) have received more attention as potential alternatives to replace antimicrobial growth promoters (AGP) in animal production due to their known biological properties: antimicrobial, antioxidant and anti-inflammatory^16,17^. A few studies have reported that some EOs can suppress pathogenic bacteria while stimulating beneficial microorganisms such as *Lactobacillus* in the pig gut^18,19^. Specifically, the citrus EOs, which are by-products of orange juice production^20^, could be an excellent alternative for that purpose since they have shown good potential to fight pathogenic bacteria such as *Listeria* spp.^21^, *Salmonella* spp.^22^, *E. coli*, *Staphylococcus aureus* and *Bacillus cereus*^23^. Furthermore, the use of citrus EOs in animal feed could become feasible since there is a huge availability of these oils in the worldwide market. Therefore, the aim of this study was to evaluate the selective antibacterial activity of six commercial citrus EOs on ETEC strains isolated from pig gut and on two *Lactobacillus* species belonging to ATCC, as well as to determine the chemical composition of these citrus EOs.

## Results

### Antibacterial activity

#### Screening by disc diffusion

**Figure 1:**
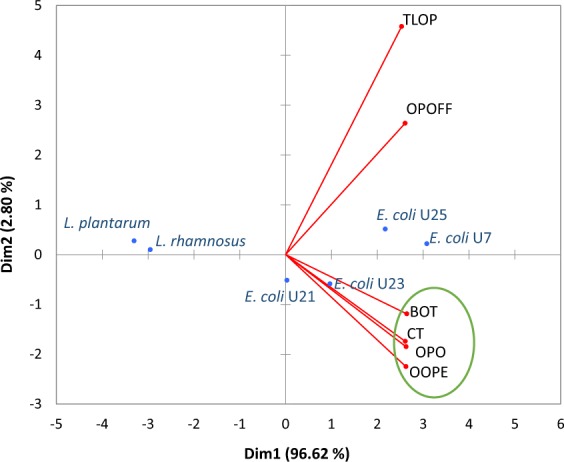
Principal component analysis (PCA) of six citrus EOs based on their antibacterial activity on ETECs strain and *Lactobacillus* species. OOPE = Orange oil phase essence, OPO = Orange peel oil, BOT = Brazilian orange terpenes, TLOP = Tahiti lime oil phase, OPOFF = Orange peel oil five fold and CT = Citrus terpenes.

The antibacterial activity screening of the six citrus EOs on the ETECs and *Lactobacillus* species is shown in Table 1. The association of inhibition zone diameter (IZD) means of six citrus EOs, when evaluated on ETECs and *Lactobacillus* species, by principal component analysis (PCA), showed that the first principal component explained 96.62%, and the second component 2.80% of the total variance (Fig. 1). Therefore, a good representation of antibacterial activity of these citrus oils was obtained. The IZD data showed that all the citrus oils had high antibacterial activity on all the ETECs, while low activity on the two *Lactobacillus* species was observed (Table 1). Consequently, it is possible to highlight these citrus oils as having a selective antibacterial activity. Furthermore, in contrast to the antibiotic colistin, an antibiotic that presented a selective performance (antibacterial activity on ETECs and no activity on *Lactobacillus* species), the citrus EOs showed superior performance on ETECs. Looking at the PCA (Fig. 1), four of the six citrus EOs, BOT, OOPE, CT and OPO, were the most selective oils since a closer association of their antibacterial activity with ETECs than with *Lactobacillus* species was observed. IZDs of these oils on ETECs were >18 mm. However, from these four citrus EOs, BOT stood out by exhibiting the best selective antibacterial activity (*p* < *0.05*), since it presented the largest IZDs to all ETECs and considerable low IZDs (*p* < *0.05*) to the two evaluated *Lactobacillus* species. Therefore, this oil was selected to continue the study.

**Table 1:** Antibacterial activity of the citrus essential oils on ETECs strains isolated from pig gut and *Lactobacillus* strains*.

Strain		*E. coli* U7			*E. coli* U21			*E. coli* U23			*E. coli* U25			*L. rhamnosus* ATCC 7469			*L. plantarum* ATCC 8014		
Essential oil^***^		IZD^**^	PC^**^	% I	IZD^**^	PC^**^	% I	IZD^**^	PC^**^	% I	IZD^**^	PC^**^	% I	IZD^**^	PC^**^	% I	IZD^**^	PC^**^	% I
1	BOT	27.4 ± 0.8^ab,A^	17.4 ± 0.2	157.4	20.1 ± 0.3^a,C^	12.4 ± 0.3	161.6	22.8 ± 0.9^a,B^	13.3 ± 0.2	171.0	25.4 ± 1.5^a,AB^	15.6 ± 0.7	162.4	9.9 ± 0.3^bc,A^	6.0 ± 0.0	165.7	8.8 ± 0.4^ab,B^	6.0 ± 0.0	161.6
2	TLOP	24.3 ± 1.1^bc,A^	15.3 ± 1.8	158.7	12.5 ± 1.7^c,B^	12.4 ± 0.4	100.8	14.9 ± 0.8^b,B^	12.9 ± 0.1	115.6	22.3 ± 2.5^a,A^	16.7 ± 1.2	133.9	8.5 ± 0.2^d,A^	6.0 ± 0.0	141.3	8.0 ± 0.3^b,A^	6.0 ± 0.0	133.9
3	OPO	23.8 ± 1.4^c,A^	16.1 ± 1.8	148.2	18.1 ± 0.2^b,B^	12.5 ± 0.5	144.6	20.8 ± 1.6^a,AB^	12.8 ± 0.4	162.9	22.2 ± 1.6^a,AB^	16.4 ± 0.3	135.6	9.1 ± 0.3 ^cd,A^	6.0 ± 0.0	151.8	8.4 ± 0.4^ab,B^	6.0 ± 0.0	140.2
4	OPOFF	13.4 ± 0.9^d,A^	17.1 ± 0.5	78.3	9.3 ± 0.9^d,B^	12.7 ± 0.1	73.2	10.1 ± 0.4^c,B^	12.6 ± 0.1	80.2	13.3 ± 0.9^b,A^	15.6 ± 1.2	85.4	6.0 ± 0.0^e,A^	6.0 ± 0.0	100.0	6.0 ± 0.0^c,A^	6.0 ± 0.0	100.0
5	OOPE	25.7 ± 0.6^abc,A^	16.9 ± 0.6	151.1	19.2 ± 0.5^ab,B^	12.6 ± 0.5	152.4	22.3 ± 1.5^a,AB^	13.0 ± 0.5	172.7	22.9 ± 1.3^a,A^	15.8 ± 0.4	144.7	10.4 ± 0.5^ab,A^	6.0 ± 0.0	173.2	8.8 ± 0.1^ab,B^	6.0 ± 0.0	146.8
6	CT	28.6 ± 2.2^a,A^	17.7 ± 1.1	161.9	19.8 ± 0.8^ab,B^	12.1 ± 0.6	163.0	22.1 ± 1.2^a,B^	12.8 ± 0.7	172.4	22.5 ± 0.7^a,B^	15.8 ± 0.5	142.0	10.9 ± 0.5^a,A^	6.0 ± 0.0	182.4	9.1 ± 0.4^a,A^	6.0 ± 0.0	151.0

***OOPE = Orange oil phase essence, OPO = Orange peel oil, BOT = Brazilian orange terpenes, TLOP = Tahiti lime oil phase, OPOFF = Orange peel oil five fold and CT = Citrus terpenes.

**IZD: Inhibition zone diameter; PC: Positive control (Colistin); %I: inhibition of essential oil in relation to colistin.

*Values are means ± Standard Deviation (SD) of triplicate determinations expressed in mm including 6 mm of paper disk.

^a,b^Mean values within a column having different superscripts are significantly different (essential oils) by the least significant difference Tukey test (p < 0.05).

^A,B^Mean values within a row having different superscripts are significantly different (strains) by the least significant difference Tukey test (p < 0.05).

Regarding the susceptibility of ETECs, *E. coli* U7 was the most sensitive strain (*p* < *0.05*) to the activity of the citrus EOs, since the largest IZDs were observed for this ETEC. Conversely, *E. coli* U21 was the least sensitive or the most resistant ETEC, since were gotten the lowest IZDs (*p* < *0.05*) on this bacterium. In the case of *Lactobacillus* species, it was observed that *L. plantarum* was the more resistant beneficial bacterium to the citrus EOs activity, since lowest IZDs were gotten on this bacterium. This behavior of the sensitivity for the bacteria tested is also represented in the PCA (Fig. 1), where the farthest association of *E. coli* U21 to the EOs is observed, and the closer association of *L. rhamnosu*s than of *L. plantarum* to EOs can be observed. Therefore, *E. coli* U21 was selected as the most resistant ETEC and *L. rhamnosus* selected as the most sensitive beneficial bacterium to the antibacterial activity of the citrus oils.

#### Determination of minimal inhibitory concentration (MIC) and minimal bactericidal concentration (MBC)

**Figure 2:**
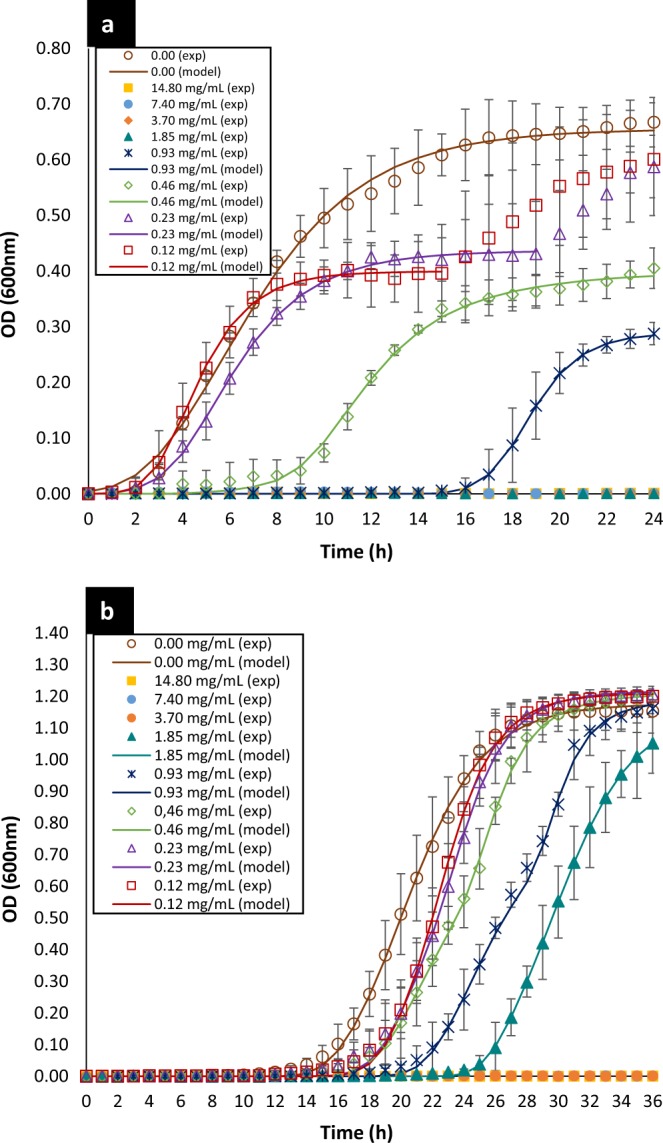
Bacterial growth kinetic as function of BOT concentrations of *E. coli* U21 (**a**) and *L. rhamnosus* (**b**). The dots are the experimental values; the vertical bars are the standard deviation and the curves are the modified Gompertz model (Eq. (6)). BOT = Brazilian orange terpenes

The MIC and MBC determinations were made only using the most selective EO, which was BOT, the most resistant *E. coli* strain (*E. coli* U21), and most sensitive *Lactobacillus* species (*L. rhamnosus*), as determined in the screening phase. The MIC and MBC values for BOT, as determined by survival curves and resazurin test, are shown in Table 2. The MIC for *E. coli* U21 was 1.85 mg/mL (Fig. 2a), which was also the MBC for this bacterium. The MIC for *L. rhamnosus* was 3.70 mg/mL (Fig. 2b) and the MBC was 7.40 mg/mL (Table 2). Thus, these results reaffirm the selective antibacterial activity of this citrus oil, since to totally inhibit the growth of the beneficial bacterium *L. rhamnosus* required an EO concentration equivalent to twice the MIC observed for *E. coli* U21, and to kill it necessitated a concentration of four times the *E. coli* U21 MIC. Therefore, pathogenic bacterium was more sensitive to BOT than beneficial bacterium.

**Table 2:** MIC and MBC for Brazilian orange terpenes (BOT).

Bacterial strain	Brazilian Orange Terpens	
	MIC (mg/mL)^a^	MBC (mg/mL)
*E. coli* U21	1.85	1.85
*L. rhamnosus* ATCC 7469	3.70	7.40

^a^Determined by survival curves and resazurin test.

#### Bacterial growth modeling and calculation of kinetic parameters

The curves representing the bacterial growth kinetic for *E. coli* U21 and *L. rhamnosus*, when they were exposed to different concentrations of the BOT, are shown in Fig. 2. The Gompertz model modified by Zwietering *et al*.^24^ was used to fit the data of the bacterial growth (Eq. 6). The parameters obtained of this model for both bacteria are shown in Table 3.

**Table 3:** Calculated parameters* of Modified Gomperzt Model (Eq. (6)^24^) for each evaluated concentration of Brazilian orange terpenes (BOT).

C_EO_^c^ (mg/mL)	*E. coli* U21				*L. rhamnosus* ATCC 7469			
	A (OD_600 nm_)^a^	µ_max_ (h^−1^)^b^	λ (h)^a^	R^2^	A (OD_600 nm_)^b^	µ_max_ (h^−1^)^b^	λ (h)^a^	R^2^
14.80	—	—	—	—	—	—	—	—
7.40	—	—	—	—	—	—	—	—
3.70	—	—	—	—	—	—	—	—
1.85	—	—	—	—	1.143 ± 0.027	0.165 ± 0.005	26.61 ± 2.19	0.99
0.925	0.289 ± 0.016	0.078 ± 0.004	16.88 ± 0.97	0.99	1.192 ± 0.047	0.215 ± 0.104	23.85 ± 3.95	0.99
0.463	0.400 ± 0.054	0.058 ± 0.018	8.16 ± 1.31	0.99	1.210 ± 0.040	0.197 ± 0.089	20.24 ± 3.37	0.99
0.231	0.437 ± 0.060	0.072 ± 0.008	3.04 ± 0.57	0.98	1.216 ± 0.032	0.202 ± 0.048	19.55 ± 2.34	0.99
0.116	0.400 ± 0.053	0.097 ± 0.006	2.58 ± 0.45	0.98	1.210 ± 0.028	0.204 ± 0.035	19.35 ± 1.77	0.99
0.00	0.655 ± 0.049	0.073 ± 0.012	2.30 ± 0.55	0.99	1.176 ± 0.054	0.148 ± 0.008	16.61 ± 1.94	0.99

*A = maximal bacterial culture density (OD600 nm), µ_max_ = the maximum specific growth rate (h^−1^), λ = the lag phase duration (h).

(−)Undetermined parameters due to total inhibition.

^c^Concentration of essential oil.

^a^There are significant differences in the growth kinetics parameters after exposure to essential oil concentrations (p < 0.05).

^b^No significant differences were observed (p < 0.05).

The survival curves or growth kinetics of *E. coli* U21 (Fig. 2a) showed that the four highest EO concentrations totally inhibited the growth of this bacterium and the growth was only observed for concentrations equal to or under 0.925 mg/mL of BOT oil. In the case of *L. rhamnosus* (Fig. 2b), bacterial growth was observed up to the concentration of 1.85 mg/mL of BOT and the three highest concentrations caused complete inhibition of this bacteria. Furthermore, we observed that EO concentration was able to provoke higher disturbances on the normal growth kinetic of *E. coli* U21 than *L. rhamnosus*. The modified Gompertz model allowed us to evaluate these disturbances with more accuracy through the three main biological parameters that it considers: maximal bacterial culture density (A), maximum specific growth rate (µ_max_) and lag phase duration or adaptation time (λ).

**Figure 3:**
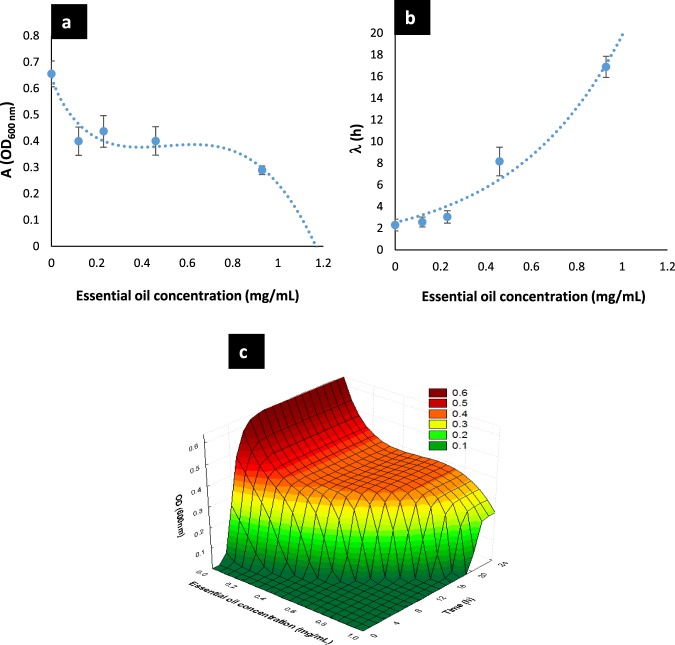
Parameters *A* (**a**) and λ (**b**) of the modified Gompertz model (Eq. (6)) to *E. coli* U21 as function of the BOT concentration. The dots are the experimental values; the vertical bars are the standard deviation and the curves are the model of Eqs. (1) and (2), respectively. (**c**) General model that describes the bacterial culture density of *E. coli* U21 as function of the time of treatment and the BOT concentration applied (Eq. (4)). BOT = Brazilian orange terpenes.

The values of parameter *A* were significantly affected (*p* < *0.05*) for *E. coli* U21, since *A* was greatly reduced as the EO concentration was increased (Table 3). For instance, *A* was reduced in 38.9% at the lowest concentration and in 55.9% at the highest subinhibitory concentration (0.925 mg/mL), in contrast with the control (0 mg/mL of BOT). The parameter *A* had an inverse sigmoidal behavior as function of the EO concentration (Fig. 3a) and the mathematical function that describes this behavior to *E. coli* U21 is shown in Eq. (1), which had a good fit (R^2^ = 0.91). Regarding *L. rhamnosus*, this parameter was not affected by EO concentrations (Table 3), no significant differences were detected (*p* < *0.05*), and it was considered the average in the general model of this bacterium, which was *A*_*LR*_ = 1.191 ± 0.028.

1 $${A}_{EC}=3.47+4.46{({C}_{EO})}^{1.5}-\mathrm{2.83.}{e}^{({C}_{EO})}$$

The maximal growth rate, that is, the parameter µ_max_ for both bacteria, *E. coli* U21 and *L. rhamnosus*, was not affected by the EO concentrations since no significant differences were detected among the concentrations tested (*p* < *0.05*). Consequently, it was considered an average growth rate of each bacterium for the general model, 0.076 ± 0.014 h^−1^ to *E. coli* U21 and 0.189 ± 0.026 h^−1^ to *L. rhamnosus*. As observed, the growth rate was quite higher for *L. rhamnosus* than *E. coli* U21.

**Figure 4:**
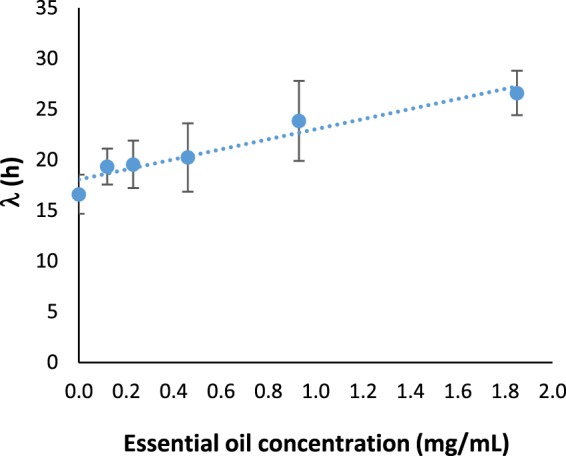
Parameters λ of the modified Gompertz model (Eq. (6)) to *L. rhamnosus* as function of the BOT concentration. The dots are the experimental values; the vertical bars are the standard deviation and the curves are the model of Eq. (3). BOT = Brazilian orange terpenes.

Regarding parameter λ, which is the lag phase duration, we observed that for both bacteria this parameter increased as the EO concentration was increased. For *E. coli* U21, λ was slightly longer than control at the three lowest EO concentrations, but from 0.463 mg/mL to above, the lag phase was notably increased. For instance, λ increased approximately eight times at the highest subinhibitory concentration of BOT oil (0.925 mg/mL), in contrast with the control (0.00 mg/mL). Therefore, this parameter had an exponential behavior as function of the EO concentration (Fig. 3b) and the mathematical function that describes it is shown in Eq. (2), which had a good fit (R^2^ = 0.97). For *L. rhamnosus*, λ only suffered a significant increase, approximately 1.6 times at the highest effective subinhibitory BOT concentration (1.85 mg/mL). The behavior of λ to this beneficial bacterium was lineal as function of the EO concentration (Fig. 4); the mathematical function that describes it is shown in Eq. (3) and it had a good fitting (R^2^ = 0.93).

2 $${\lambda }_{EC}=2.52{e}^{2.06({C}_{EO})}$$

3 $${\lambda }_{LR}=4.971{C}_{EO}+18.06$$

Finally, the modified Gompertz model to describe the bacterial growth kinetics as function of EO concentration (0.00 < C_EO_ < 14.80 mg/mL) and time of exposure (in hours), is shown in Eq. (4) for *E. coli* U21, and in Eq. (5) for *L. rhamnosus*. Additionally, the model for *E. coli* U21 was plotted in 3D and is shown in Fig. 3c. The surface obtained highlights the shifts in the bacterial growth kinetic of *E. coli* U21 as function of initial EO concentration.

4 $${y}_{EC(t,{C}_{EO})}=(3.47+4.46{({C}_{EO})}^{1.5}-\mathrm{2.83.}{e}^{({C}_{EO})})\,\exp \,(-\exp (\frac{0.076e(2.52{e}^{2.06({C}_{EO})}-t)}{3.47+4.46{({C}_{EO})}^{1.5}-\mathrm{2.83.}{e}^{({C}_{EO})}}+1))$$

5 $${y}_{LR(t,{C}_{EO})}=1.191\,\exp (-\exp (\frac{0.1896e(4.971{C}_{EO}+18.06-t)}{1.191}+1))$$

Therefore, evaluation of growth bacterial kinetic parameters confirmed that the citrus oil, BOT, had a stronger effect on the pathogenic bacterium than on the beneficial bacterium, provoking higher disturbances in its growth kinetics. This proves the selective antibacterial features of the BOT oil.

### Chemical composition characterization of essential oils

**Figure 5:**
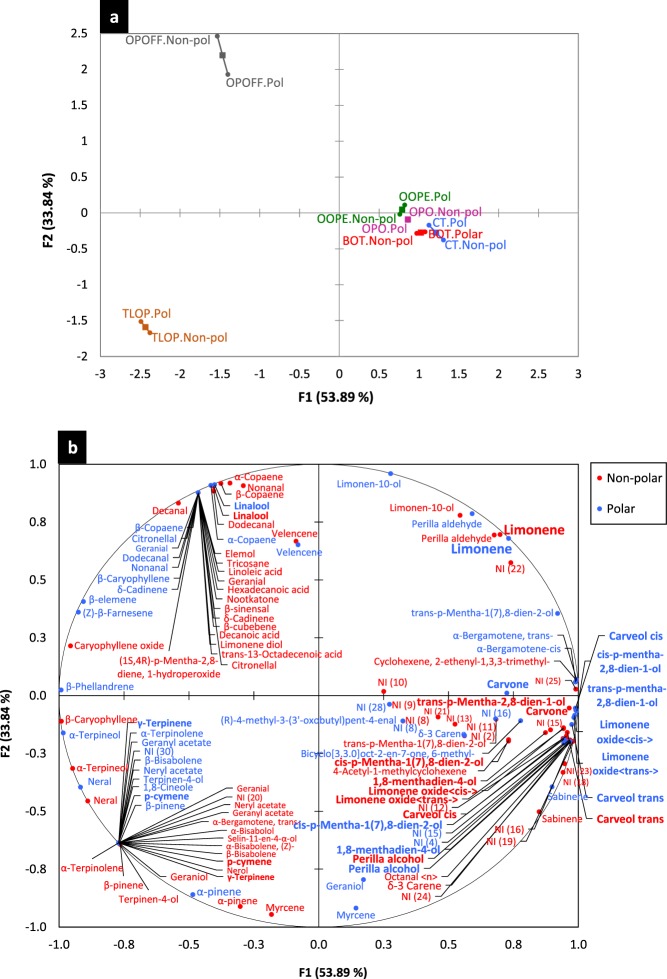
Multiple factor analysis (MFA) of the chemical composition profile of six citrus commercial EOs. The individual factor map of the overall chemical composition profiles by polar and non-polar identification (**a**) and biplot of the detailed chemical composition profile (**b**). OOPE = Orange oil phase essence, OPO = Orange peel oil, BOT = Brazilian orange terpenes, TLOP = Tahiti lime oil phase, OPOFF = Orange peel oil five fold and CT = Citrus terpenes.

The chemical composition of the six citrus EOs is shown in Table S1 (Supplementary information). Overall, the identification of the chemical composition of each citrus EO by both columns was quite similar, as is observed in the individual factor map of the MFA (Fig. 5a), the polar and non-polar identification points were very close to each other. In addition, a good representation of the chemical composition data was obtained, since the first dimension of the MFA explained 53.89% and the second dimension explained 33.84% of the total variance. For the six citrus oils, limonene was detected as the major compound; however, TLOP had a negative association with this compound in both dimensions (Fig. 5b), since it presented the lowest relative amount of limonene (46.53%/47.46%).

Moreover, it was observed that BOT, CT, OOPE and OPO had a positive association with limonene in the two dimensions of the MFA and OPOFF had that positive association in the second dimension, showing all these EOs presented higher amount of limonene (Fig. 5b). However, OPOFF presented a fully different chemical profile than BOT, CT, OOPE and OPO oils (Fig. 5a) and it was the oil that presented the weakest antibacterial activity. BOT, CT, OOPE and OPO oils presented a very close chemical composition profile and they were characterized by having the highest selective antibacterial activity. Therefore, this allows us to suggest that the antibacterial activity of the citrus EOs could not be attributed specifically to limonene. Minor common compounds such as cis-limonene oxide, trans-carveol, carvone, trans-limonene oxide, cis-p-Mentha-2,8-dien-1-ol, trans-p-Mentha-2,8-dien-1-ol, perrilla alcohol, cis-carveol, cis-p-Mentha-1(7),8-dien-2-ol and 1,8-menthadien-4-ol (Fig. 5b) were detected in these four citrus EOs and they were either present exclusively in these four EOs or in higher amounts than TLOP and OPOFF oils. Therefore, these minor compounds could be implicated to confer the selective and highest antibacterial activity of BOT, CT, OOPE and OPO oils.

In addition. we observed that TLOP and OPOFF had a different chemical profile. The TLOP profile is shown in the third quadrant of the MFA (Fig. 5b); besides limonene, this oil had a high amount of p-cymene and γ-terpinene. The OPOFF profile is shown in the second quadrant of the MFA (Fig. 5b), and linalool was detected as second major compound in it.

## Discussion

Citrus EOs are one of the main by-products of the orange juice industry and could have several applications besides cosmetology and the food industry. Citrus oils have been reported as having antimicrobial properties^20–22,25^, but testing their antimicrobial activity on bacteria affecting animal farms has not been greatly mentioned. *E. coli* causing post-weaning diarrhea in piglets during their early life is a critical problem for the pig industry^2^. The antibacterial activity of commercial citrus EOs evaluated in this study on *E. coli* strains isolated from pig gut and on *Lactobacillus* bacteria showed that, in overall, citrus EOs presented a selective antibacterial activity, having higher activity on pathogenic bacteria *E. coli* than on beneficial bacteria *Lactobacillus* spp. The selectivity towards bacterial pathogens by EOs or EO compounds rather than beneficial bacteria have been already reported^18,26,27^, EOs or single EO compounds were shown to have higher inhibitory effect on *E. coli* O157:H7, *E. coli* K88, *S. typhimurium* than on *Lactobacillus* and *Bifidobacterium* spp^18^. As an alternative to AGPs, the selectivity of citrus oils could be an important feature of antimicrobial spectrum, which commonly has not been considered to conventional AGPs long used, since the aim is to have an effect on the pig gut. Thus, current search for antimicrobial substances as alternatives to AGPs should consider selectivity aspects between pathogenic and beneficial gut bacteria. The lower effect on beneficial bacteria by an AGP and its potential alternatives (such as citrus EOs) would be desirable since beneficial bacteria such as *Lactobacillus* spp. contribute to fighting pathogen colonization in the gut and preventing gut infections. Thereby, reinforcing gut microbiota and contribute to improve animal health^27^.

The capability of citrus EOs to inhibit pathogenic bacteria has been well reported in several studies. For instance, tangerine EO (*Citrus reticulata*) was reported as having an inhibitory effect on *S. aureus*, *Bacillus subtilis*, and *Pseudomonas aeruginosa*^28^. Another study proved the high effectiveness of the lemon EO (*Citrus limon* L. Burm) to inhibit several strains of *Listeria monocytogenes*, *S aureus* and *Salmonella enterica* associated with foodborne diseases^25^ Also, it has been reaffirmed the anti-salmonella^22^ and anti-listeria^21^ activities of several commercial citrus oils. Specifically, the antibacterial activity of citrus oils on *E. coli* has been investigated. Tangerine EO (*Citrus reticulata*) was shown to be effective to produce an inhibition of 14.6 ± 1.1 mm on *E. coli*^28^. A total inhibition of *E. coli* growth by this oil was found at 1.96 mg/mL^29^. A close value to this MIC was found for BOT oil in our study (1.85 mg/mL = 0.21%v/v). Other citrus EOs such as bergamot, orange and lemon were also effective to produce inhibitions ≥18 mm on *E. coli* O157, but full inhibition of the growth of this bacterium was reported at higher MICs than the BOT oil, between 0.5–1.0%v/v^30^. Similarly, another study reported higher MICs than the BOT to mandarin and lemon EOs on *E. coli*, 5 and 30 µL/mL, respectively^31^. In addition, the EO of sweet orange (*Citrus sinensis* Osbeck) has presented an inhibitory effect on *E. coli* at 18.8 µL/mL^32^, also considered as a high MIC in contrast to the BOT oil. Conversely, the non-effectiveness of several citrus oils to fight *E. coli* affecting animals, such as *E. coli* associated with poultry colibacillosis, has been reported^33^. In comparison, the citrus EOs tested in our study were quite effective in treating *E. coli* affecting pigs.

Furthermore, some studies highlighted that citrus EOs have higher effectiveness to inhibit Gram-positive pathogenic bacteria than Gram-negative pathogenic bacteria^30,34^. However, in our study, the opposite was observed, since the Gram-negative *E. coli* was more sensitive than the Gram-positive *Lactobacillus* spp. to the activity of the citrus oils. The difference in the sensitivity to EOs between these two groups of bacteria has been hypothesized to be the consequence of differences in the cell wall structure, since Gram-positive bacteria lack an outer membrane (OM), which Gram-negative bacteria have. This OM contains lipopolysaccharides (LPS) with polar ends (O-polysaccharides) and transmembrane channels (porins), which permit the passage of hydrophilic solutes and make difficult for hydrophobic compounds to diffuse such as EOs components into the cell. Therefore, this would allow Gram-negative bacteria be more resistant to EOs^35^. Nonetheless, the antibacterial spectrum of EOs depends on the specificity of the functional groups of EO compounds to single or multiple targets. Some EO compounds have the ability to disintegrate the OM of Gram-negatives as *E. coli*, release the material associated to this membrane and penetrate the cell, provoking a disruptive effect^36^. Probably, the compounds present in citrus EOs may have this ability due to their higher effectiveness observed on this Gram-negative bacterium, *E. coli*. On the other hand, the antibacterial activity of citrus oils on Gram-positive beneficial bacteria has been little reported. Orange, lemon, mandarin and grapefruit EOs had a low inhibitory effect on *Lactobacillus sakei* and *Lactobacillus curvatus*, exhibiting the orange oil the lowest effect on these bacteria (12.8 ± 0.5 and 13.5 ± 0.2 mm, respectively)^37^. The authors demonstrated that the inhibitory effect of these four oils was dose-dependent causing inhibition of those *Lactobacillus* species only at the highest concentrations tested^37^. This was also noticed in our study, where the citrus oils exhibited IZDs lower than 11 mm to *Lactobacillus* species. Moreover, *L. rhamnosus* was inhibited at a high BOT concentration and killed even at an upper concentration, thus showing  BOT had a low antibacterial activity on *L. rhamnosus*. Although general structures and biosynthesis pathways among Gram-positive bacteria are conserved, some Gram-positive bacteria, such as *Lactobacillus* spp., could show low sensitivity to antimicrobials, such as EOs, since the cell wall of Gram-positive lactic acid bacteria (LAB) as *Lactobacillus* spp. possess unique properties that could confer intrinsic resistance to some antimicrobial agents^38^. For instance, the intrinsic resistance to antibiotics of some *Lactobacillus* (e.g. to vancomycin) would be related to the fact of having a D-lactate instead of D-alanine as the last amino acid in the peptidic chain of the peptidoglycan layer of their cell wall^38,39^, which would avoid the antibiotic binds to the peptidic chain and cause the inhibition of these bacteria^40^.

Additionally, it was observed that BOT oil caused higher disturbances on the growth kinetics of *E. coli* than *L. rhamnosus*, significantly affecting its maximal culture density and the lag phase duration. Both parameters were dose-affected and changed as function of the BOT concentration. The higher dose-dependent effect of some EOs and single EO compounds on the growth kinetic of *E. coli* than on *Lactobacillus* spp. has already been observed^27^. Oregano, thyme and rosemary EOs, carvacrol, eugenol and thymol provoked higher reduction on the maximal culture density of *E. coli* strains than *Lactobacillus fermentum* and *Lactobacillus reuteri* with increasing of the concentration of EOs/EO compounds^27^. Also, the dose-dependent effect of carvacrol to extend the lag phase of *E. coli* by increasing the concentration of this compound has been proved^41^. In addition, some combinations of EOs have been reported as more efficient to cause an increase of the *E. coli* lag phase. For instance, combinations of oregano with basil EOs and oregano with lemon balm EOs were able to significantly increase the *E. coli* lag phase, approximately 7.4 h and 3.6 h longer, respectively, compared to when oregano EO was used alone^42^. Regarding *L. rhamnosus*, in our study, we observed only the lag phase duration was extended as the BOT concentration was increased. This effect on *L. rhamnosus* has been previously observed with the oil of *Melaleuca armillaris*, which additionally reduced the growth rate and final culture density with increasing of the EO concentration^43^. Therefore, this oil had a higher dose-dependent effect on the growth kinetic parameters of *L. rhamnosus* than the citrus oil (BOT) tested in our study. Moreover, a recent study observed that *Eucalyptus globulus* and *Pimenta pseudocaryophyllus* EOs presented a dose-dependent effect on the lag phase of *L. rhamnosus* as well; however, *P. pseudocaryophyllus* oil caused higher extension of this parameter in comparison to *E. globulus* oil at the same sub-MICs^44^.

Limonene has been shown as a major compound of citrus EO composition and most of their biological activities have been attributed to this compound. All citrus oils evaluated in our study presented limonene as the major compound. However, the mismatching between limonene content and antibacterial activity of these oils suggested that their antibacterial activity cannot be attributed exclusively to limonene. Some studies have already reported the lack of antibacterial activity of limonene individually tested. For instance, the pompia EO (*Citrus limon var. pompia*), which presented limonene as major compound (28%), at a concentration of 256.3 mg/mL, presented an antibacterial effect on several pathogenic bacteria, but when limonene was evaluated alone, it did not exhibit any antibacterial effect^45^. Thus, this proved that limonene would not be the compound responsible for the antibacterial activity observed for this oil. Nonetheless, coexisting minor compounds in citrus oils could contribute to conferring the antibacterial property of these oils. In mandarin EO, compounds like octanal, decanal, citral, citronellal, linalool, α-sinensal and thymol were suggested as possible collaborators to the antibacterial activity, when this oil (with 56.8% of limonene) was tested against Gram-negative and Gram-positive bacteria^28^. Other minor compounds, belonging to oxygenated monoterpenes class, such as 4-terpineol, α-terpineol, cis-geraniol, β-citral, nerol and α-citral, might also be implicated in conferring the antibacterial activity of citrus oils, since they have been detected in high amounts in the composition of the citrus EO that presented high antibacterial activity^25^. Minor oxygenated monoterpenes compounds (cis-limonene oxide, trans-carveol, carvone, trans-limonene oxide and perrilla alcohol) were also detected in the group of the most selective citrus oils of our study. Possibly, these compounds might play an important role in conferring the selective antibacterial activity of citrus EOs. In addition, an orange cold pressed EO rich in limonene (85.3%) presented an antibacterial activity on *E. coli* ten times higher than limonene alone, and even minor compounds, such as linalool, pinene and terpineol, presented a higher activity than limonene^46^. Furthermore, the antibacterial activity of limonene has been shown to be variable and depending on its stereoisomeric form present in the EO. The (−) stereoisomer of limonene could inhibit *E. coli* at a lower concentration (8 mg/mL) than the (+) stereoisomer (11 mg/mL). On the other hand, limonene alone has been proved to stimulate the growth of beneficial bacteria as *L. fermentum*, instead of having any inhibitory effect on it^27^. Likewise, limonene has been reported as not effective to inhibit several *Lactobacillus* species including *L. rhamnosus* ATCC 7469^45^, the bacteria also evaluated in our study, and which showed be the more resistant to the antibacterial activity of the citrus oils proved. Therefore, it would be possible to infer that limonene could collaborate with the selective activity of citrus oils when it is present in the gut, promoting the beneficial bacteria while other minor compounds could act in inhibiting pathogenic bacteria. It has been reported that in an EO, major and minor components probably act in synergism to confer the biological properties of the EO^47^. When an EO compound is proved, individually, its effect may differ from the effect that this compound may have in combination with the other compounds inside the EO. Thus, it would be recommended the use of the whole EO instead of single EO compounds, since every compound inside an EO could exert a different mechanism of action on the bacteria cell^48^, and this could reduce the chance to bacteria develop easily resistance to the EO. Contrariwise, bacteria could develop a rapid and easy mechanism of resistance to a single EO compound, as in the case of an antibiotic, which consist of a single compound. Several mechanisms of action of EOs have been proposed in the literature. The mechanism of action comprises a serie of events on the bacterial cells. Initially, they can destabilize the cellular architecture, leading to the breakdown of membrane integrity and thus increased permeability of cellular constituents. This disrupts many cellular activities, including energy production, membrane transport, and other metabolic regulatory functions^48^. In addition, EOs can alter the membrane fatty acids composition, the membrane proton motive force and affect proteins in the cytoplasmatic membrane. Additionally, EOs can interfere with the quorum sensing activity, decreasing the proteolytic activity, biofilm formation, and virulence factors expression and their functions, as well as to affect the metabolome of bacteria^35^.

In conclusion, our study highlights as an important feature of antimicrobial spectrum the selectivity between pathogenic and beneficial gut bacteria, which should be considered when searching for antimicrobial substances as alternatives to conventional AGPs, since the aim is to have an effect on the pig gut. Our study has proved, by a screening, MIC determination, and growth kinetic parameters evaluation, the selective antibacterial activity of citrus EOs on *E. coli* and *Lactobacillus* spp., thus suggesting these EOs as potential alternatives to AGPs. Consequently, based on the selective performance and the huge viability in the global market of citrus EOs, the possible application of these oils in the pig production sector could turn feasible. In addition, chemical composition characterization showed that minor compounds present in these citrus oils would be implicated in conferring their selective activity, instead of limonene, the major present compound, playing this role exclusively. Finally, our results motivate further research to clarify, for instance, the possible mechanism of action that citrus oils would have on pathogenic and beneficial bacteria as well as their direct effect on the pig gut and on the microbiota resident in it.

## Material and Methods

### Essential oils supply

Six citrus commercial EOs were used in the study. These were by-products from orange juice production and were supplied by a factory from São Paulo State, Brazil. The oils were named by the factory as follows: Orange oil phase essence (OOPE), Orange peel oil (OPO), Brazilian orange terpenes (BOT), Tahiti lime oil phase (TLOP), Orange peel oil five fold (OPOFF) and Citrus terpenes (CT). Once the samples were received, they were kept in amber bottles under refrigeration (4 °C) until use.

### Bacterial strains

The evaluated bacterial strains in this research were four ETECs strains and two *Lactobacillus* species. The ETECs strains were isolated from pig gut and provided by The Swine Heath Laboratory of the Department of Preventive Veterinary Medicine and Animal Health from FMVZ-São Paulo University: *E. coli* U7 (K88+/LT+/STb+), *E. coli* U21 (K88+/LT+/STb+/F18+/Sta+), *E. coli* U23 (LT+/STb+/F18+) and *E. coli* U25 (LT+/STb+/F18+/Sta+). The two *Lactobacillus* species were standard cultures from the American Type Culture Collection (ATCC), *L. plantarum* ATCC 8014 and *L. rhamnosus* ATCC 7469. ETECs strains were cultivated in Tryptic Soy Agar-Difco (TSA, Difco^TM^) at 37 °C for 18–20 h and *Lactobacillus* species in MRS (Man, Rogosa and Sharpe agar, Difco^TM^) agar at 30 °C for 48 h. After activation, the bacteria were sub-cultured in Brain-Heart Infusion broth or MRS both (Difco^TM^) supplemented with 15% of glycerol. After incubation, they were stored at −20 °C until their use.

### Antibacterial activity

#### Screening by disc diffusion

All EOs were initially screened by disc diffusion method, following the standard protocol M02-A11 from the Clinical and Laboratory Standards Institute^49^. EO solutions were prepared at 90% (v/v), using acetone as an emulsifier to improve dispersion. ETECs strains (*E. coli* U7, U21, U23 and U25) were grown on TSA agar and *Lactobacillus* species on MRS agar. Isolated colonies of each bacterium were transferred to tubes containing sterile saline solution (0.85%) until reaching an optical density within 0.08 to 0.1 abs, at 625 nm, which corresponds to 0.5 McFarland standard, therefore containing ~1–2 × 10^8^ CFU/mL^49^. After this, Mueller Hinton (MH) agar plates (*E. coli*) and MRS agar plates (*Lactobacillus* spp.) were inoculated and the bacterial inoculum spread. Seven microliters of each EO solution (90% v/v) were placed on 6-mm diameter sterile paper discs (Whatman N° 3), which was transferred to the inoculated agar plates. Three discs with the same EO solution were placed in each plate; one disc of colistin (15 µg/disc) was used as a positive control, and one disc of acetone (10 µL/disc) was used as a negative control since its non-antimicrobial activity was proved. Then, the agar plates were incubated at 37 °C for 24 h (*E. coli*) and at 30 °C for 48 h (*Lactobacillus* spp.). Inhibition zone diameters (IZD) were measured after incubation with the aid of a caliper rule. The experiment was carried out in three independent replicates.

The EO presenting the highest IZDs for the four ETECs strains and the lowest IZDs for the two *Lactobacillus* species was considered as the best selective EO between pathogenic and beneficial bacteria and was thus selected for further investigation. Referring to the bacterial strains, the most resistant ETEC strain and the most sensitive *Lactobacillus* specie to the activity of EOs were selected to continue with the study.

#### Determination of minimal inhibitory concentration (MIC)

The determination of the MIC of the selected EO was performed by microdilution assay in a 96-well microplate following the standard protocol M07-A9 from the Clinical and Laboratory Standards Institute, with some modifications^50^. For the assay, the standard inoculum was prepared in sterile saline (0.85% w/v) from living colonies of the selected bacteria above contained in plates of TSA agar (*E. coli*) or MRS agar (*Lactobacillus* spp.) at the optical density equivalent to 0.5 McFarland Standard (0.08–0.13 at 625 nm) as described previously. Subsequently, this inoculum was diluted at 1:100 to obtain an inoculum of 10^6^CFU/mL (final inoculum). The EO stock solution was prepared at 29.6 mg/mL (3.29%) with MH or MRS broth using Tween 80 as emulsifier. From the stock solution, two-fold serial dilutions were made in a range from 14.80 to 0.116 mg/mL along the Y-axis of the microplate. Twenty microliters from the final inoculum were added to each well containing 180 µL of several EO concentrations, being the final volume in each well of 200 µL and bacterial population of approximately 10^5^ CFU/mL. The following controls were used: culture medium control (200 µL of MH or MRS broth); growth control (180 µL of MH or MRS broth +20 µL of inoculum); Tween 80-emulsifier control (200 µL of MH or MRS broth with Tween 80) and growth control containing the emulsifier (180 µL of MH or MRS broth with Tween 80 + 20 µL of inoculum). Finally, microplates were incubated in a microplate reader (Vitor^TM^ X3, PerkinElmer) at 37 °C for 24 h for *E. coli* and at 30 °C for 36 h for *Lactobacillus* spp.

The MIC was established as the lowest EO concentration that inhibited visible bacterial growth. The existence or not of bacterial growth was evaluated by construction of survival curves and by resazurin test at the end of the incubation period. The lowest concentration that did not produce detectable absorbance values (at 600 nm) until the end of incubation was considered as the MIC obtained by survival curves. For resazurin test, 25 µL of resazurin (R7017; Sigma-Aldrich) solution at 0.0135% m/v were used per well. Thus, after visual inspection the presence of viable cells was evidenced through a change in the resazurin color from blue resazurin to pink resofurin^51^, after further incubation at 37 °C (*E. coli*) or 30 °C (*Lactobacillus spp*.) for 1 h. Assays were carried out in triplicate in three independent replicates.

#### Bacterial growth modeling and calculation of kinetics parameters

Bacterial growth kinetics (or survival curves) for each tested EO concentration was built from absorbance readings at 600 nm of the wells of the microplate configured as above, carried out every hour during the total incubation period, 24 h for *E. coli* and 36 h for *Lactobacillus* spp. Bacterial growth kinetics were modeled using the Gompertz model modified by Zwietering *et al*.^24^ (Eq. (6)), since this model considers the three main biological parameters of bacterial growth. The data were fitted to the mathematical model with a confidence level of 95% using the Levenberg–Marquardt algorithm in Statistica 12.0 (StatSoft, Inc., Tulsa, OK, USA) software.

6 $$y=A\,\exp \,(-\exp \,(\frac{{\mu }_{max}.e}{A}(-\,t)+1))$$

where: ***y*** represents the relative population size against time, the *A*, µ_max_ and λ are the three parameters that described three phases of the bacterial growth curve^24^. The asymptote *A* is the maximal bacterial culture density (OD_600 nm_), µ_max_ represents the maximum specific growth rate (h^−1^) and it is the tangent of the log phase curve, λ is the lag phase duration (h) and is defined as the x-axis intercept of this tangent. ℯ represents the number e =  2.7183.

Finally, the goodness of fit for the model was measured based on the mean square error (MSE) and on the corrected determination coefficient (corrected R^2^) for each set of data.

#### Minimal bactericidal concentration (MBC)

The determination of MBC was performed from wells containing EO concentrations where there was no visible bacterial growth. So, an aliquot of 100 µL was taken from each well and seeded in MH or MRS agar. Plates were incubated for 24 h at 37 °C for *E. coli* and for 48 h for *Lactobacillus* spp. The MBC was defined as the lowest concentration of EO able to cause total bacterial death, represented by the visible absence of colonies on the agar plates.

### Chemical composition of essential oils

The chemical composition characterization of the EOs was performed by gas chromatography coupled with mass spectrometry (GC/MS) using non-polar and polar columns.

The analysis on non-polar column was carried out using an Agilent Technology gas chromatograph 6890 Plus Series (Santa Clara, CA, USA) coupled to a selective Mass Spectrometry Detector 5973 and an Auto Sampler 7893. A fused-silica capillary column DB-5MS (J&W Scientific, Folsom, CA, USA) of 60 m × 0.25 mm id × 0.25 μm of film thickness coated with 5%-phenyl polydimethylsiloxane was used. The oven temperature was set as follows: initial oven temperature was held at 45 °C for 5 min, then raised to 150 °C at 4 °C/min for 2 min, one more time raised to 250 °C at 5 °C/min, and finally to 300 °C at 10 °C/min, which was kept for 60 min. The injector temperature was 250 °C, 2.0 µL of samples diluted in dichloromethane was injected in the “split” mode at a ratio of 30:1. EIMS, electron energy was 70 eV. The mass detector operated in full scan mode in the range of 40 to 350 m/z. The temperature of the ion source and transfer line was 230 °C and 285 °C, respectively. Helium gas was used as the carrier gas with an inlet pressure of 16.97 psi. The retention index (RI) was calculated for all the volatile compounds using a homologous series of C7–C30 n-alkanes (49451-U Sigma-Aldrich), according the linear equation of Van den Dool and Kratz^52^.

The analysis on polar column was carried out using an Agilent Technology gas chromatograph 7890^a^ Plus Series (Palo Alto, CA, USA) coupled to a selective Mass Spectrometry Detector 5975 C. A fused-silica capillary column DB-WAX (J&W Scientific, Folsom, CA, USA) of 60 m × 0.25 mm i.d × 0.25 μm of film thickness coated with polyethylene glycol was used. The oven temperature was set as follows: initial oven temperature was held at 50 °C for 5 min, then raised to 150 °C at 4 °C/min for 7 min and finally to 230 °C at 4 °C/min, which was kept for 40 min. The injector temperature was 250 °C, 2.0 µL of samples diluted in dichloromethane was injected in the “split” mode at a ratio of 30:1. EIMS, electron energy was 70 eV. Helium gas was used as the carrier gas with an inlet pressure of 16.91 psi. Mass detector operated in full scan mode in the range of 40 to 350 m/z. The retention index (RI) was calculated for all the volatile compounds as described above.

The identification of the components was performed by comparing their RI and mass spectra with data published in the literature^53,54^ and in the computer libraries (NIST 107 and WILEY 8).

### Data analysis

The IZD data from the initial screening was evaluated by analysis of variance (ANOVA) followed by the Tukey test for pairwise comparison at 5% of significance using XLSTAT (Addinsoft, New York, NY, USA). Based on this, the susceptibility of ETECs strains and *Lactobacillus* species was determined. Also, principal component analysis (PCA) using the correlation matrix was performed based on IZDs means using XLSTAT (Addinsoft, New York, NY, USA).

Furthermore, an ANOVA to detect significant differences in the growth kinetics parameters *A*, λ and µ_max_ of *E. coli* and *Lactobacillus* spp. after exposure to EO concentrations was performed (*p* < *0.05*) using R software. If significant differences were detected in those parameters, as effect of the EO concentrations, the behavior of each parameter was modeling by nonlinear regression.

In addition, the multiple factor analysis (MFA) was performed on polar and non-polar data (two tables) to describe and contrast the chemical composition profile of the six citrus EOs obtained by GC-MS, with this analysis running in the XLSTAT software.

## Supplementary information

Table S1. Chemical composition of the six citrus essential oils*.
