# Supplementary material for: Unraveling the selective antibacterial activity and chemical composition of citrus essential oils
Source: Sci Rep. 2019 Nov 27;9:17719. doi: 10.1038/s41598-019-54084-3 (PMC6881395; doi:10.1038/s41598-019-54084-3)
Supplement: Supplementary file 1 — Table S1. Chemical composition of the six citrus essential oils*. [file 41598_2019_54084_MOESM1_ESM.docx]

**Unraveling the selective antibacterial activity and chemical composition of citrus essential oils**

Carmen M. S. Ambrosio^1,*^, Natália Y. Ikeda^1^, Alberto C. Miano^2^, Erick Saldaña^1^, Andrea M. Moreno^3^, Elena Stashenko^4^, Carmen J. Contreras-Castillo^1^, Eduardo M. Da Gloria^1, *^.

^1^Department of Agri-Food Industry, Food and Nutrition, “Luiz de Queiroz” College of Agriculture, University of São Paulo, SP, Brazil.

^2^ Faculty of Engineering, Private University of the North (UPN), Trujillo, Perú

^3^School of Veterinary Medicine and Animal Science, University of São Paulo, SP, Brazil.

^4^Research Center of Excellence CENIVAM, CIBIMOL, Industrial University of Santander, Bucaramanga, Colombia.

**Table S1.** Chemical composition of the six citrus essential oils*.

| **Compounds^1^** | **LRI_c_^2^** | | **LRI_L_^3^** | | **BOT %^6^** | | **CT %^6^** | | **OOPE %^6^** | | **OPO %^6^** | | **TLOP %^6^** | | **OPOFF %^6^** | |
| --- | --- | --- | --- | --- | --- | --- | --- | --- | --- | --- | --- | --- | --- | --- | --- | --- |
|  | **NP^4^** | **P^5^** | **NP** | **P** | **NP** | **P** | **NP** | **P** | **NP** | **P** | **NP** | **P** | **NP** | **P** | **NP** | **P** |
| NI | 922 | - | - | - | - | - | - | - | - | - | 0.08 | - | - | - | - | - |
| Tricyclene | 923 | - | 923.2 | - | - | - | 0.06 | - | - | - | - | - | - | - | - | - |
| α-Thujene | 927 | - | 931 | - | - | - | - | - | - | - | - | - | 0.35 | - | - | - |
| α-pinene | 936 | 1020 | 939 | 1025.4 | 0.45 | 0.73 | 0.87 | 1.07 | 0.60 | 0.75 | 0.60 | 0.85 | 1.52 | 2.51 | - | 0.17 |
| Sabinene | 975 | 1118 | 976 | 1122 | 0.32 | 0.47 | 0.38 | 0.48 | 0.35 | 0.44 | 0.35 | 0.52 | 0.16 | 0.24 | - | 0.14 |
| β-pinene | 981 | 1106 | 980 | 1100 | 0.10 | 0.13 | 0.10 | - | 0.09 | - | - | - | 8.45 | 10.73 | - | - |
| Myrcene | 989 | 1158 | 991 | 1160.2 | 0.85 | 1.41 | 0.85 | 1.22 | 0.86 | 1.12 | 0.97 | 1.38 | 1.17 | 1.48 | 0.58 | 0.77 |
| Octanal | 1002 | 1285 | 1002.8 | 1287.2 | 0.20 | 0.01 | 0.16 | - | 0.14 | - | 0.18 | - | - | - | - | - |
| α-Terpinene | 1003 | - | 1018 | - | - | - | - | - | - | - | - | - | 0.09 | - | - | - |
| δ-3 Carene | 1011 | 1146 | 1011 | 1146.8 | 0.11 | 0.14 | 0.15 | 0.17 | 0.12 | - | 0.11 | - | - | - | - | - |
| NI | 1028 | - | - | - | 0.29 | - | 0.30 | - | - | - | - | - | - | - | - | - |
| p-cymene | 1028 | 1267 | 1024.3 | 1270 | - | - | - | - | - | - | - | - | 11.82 | 12.01 | - | - |
| β-Phellandrene | - | 1208 | - | 1209.3 | - | 0.10 | - | 0.08 | - | 0.09 | - | 0.13 | - | 0.34 | - | 0.28 |
| 1,8-Cineole | - | 1211 | - | 1211.1 | - | - | - | - | - | - | - | - | - | 0.21 | - | - |
| Limonene | 1037 | 1202 | 1031 | 1198.2 | 78.65 | 79.38 | 74.23 | 79.89 | 76.68 | 80.61 | 79.27 | 81.18 | 46.53 | 47.46 | 79.95 | 82.07 |
| γ-Terpinene | 1062 | 1243 | 1062 | 1245 | - | - | - | - | - | - | - | - | 7.65 | 8.19 | - | - |
| α-Terpinolene | 1088 | 1280 | 1088 | 1282 | - | - | - | - | - | - | - | - | 0.55 | 0.55 | - | - |
| Linalool | 1099 | 1538 | 1098 | 1543.3 | 0.28 | 0.36 | 0.31 | 0.34 | 0.85 | 0.86 | 0.59 | 0.81 | 0.36 | 0.39 | 2.37 | 3.02 |
| Nonanal | 1104 | 1390 | 1102 | 1391.5 | - | - | - | - | 0.10 | - | 0.11 | - | - | - | 0.29 | 0.27 |
| NI | 1104 | - | - | - | - | - | 0.06 | - | - | - | - | - | - | - | - | - |
| Fenchol | - | 1580 | - | 1588 | - | - | - | - | - | - | - | - | - | 0.10 | - | - |
| trans-p-Mentha-2,8-dien-1-ol | 1125 | 1622 | 1123 | 1639 | 0.89 | 2.08 | 1.14 | 1.97 | 0.98 | 1.73 | 0.71 | 1.75 | - | 0.20 | 0.20 | 0.56 |
| NI | - | 1633 | - | - | - | 0.19 | - | 0.15 | - | 0.14 | - | 0.14 | - | - | - | - |
| cis-p-Mentha-2,8-dien-1-ol | - | 1664 | - | 1652.1 | - | 1.39 | - | 1.62 | - | 1.40 | - | 1.37 | - | 0.15 | - | 0.41 |
| 4-Acetyl-1-methylcyclohexene | 1134 | - | 1139.3 | - | 0.11 | - | 0.13 | - | - | - | 0.09 | - | - | - | - | - |
| cis-Limonene oxide | 1137 | 1446 | 1134 | 1450.5 | 1.95 | 1.89 | 2.38 | 2.13 | 1.97 | 1.69 | 1.43 | 1.64 | 0.36 | 0.33 | 0.50 | 0.61 |
| trans-Limonene oxide | 1140 | 1459 | 1139 | 1461.6 | 1.64 | 0.90 | 2.17 | 1.08 | 1.73 | 0.89 | 1.32 | 0.84 | 0.30 | 0.22 | 0.42 | 0.33 |
| NI | 1143 | 1466 | - | - | - | - | - | - | - | - | - | - | 0.20 | 0.12 | - | - |
| NI | 1150 | - | - | - | - | - | 0.08 | - | - | - | - | - | - | - | - | - |
| Citronellal | 1152 | 1475 | 1153 | 1475.3 | - | - | - | - | - | - | - | - | - | - | 0.32 | 0.34 |
| NI | 1153 | - | - | - | - | - | 0.08 | - | - | - | - | - | - | - | - |  |
| Octanoic Acid | 1164 | - | 1177 | - | - | - | 0.18 | - | - | - | - | - | - | - | - | - |
| 2-cyclohexen-1-one, 4-(1-methylethenyl)- | 1172 | - | - | - | - | - | 0.14 | - | - | - | - | - | - | - | - | - |
| Nonanol | - | 1650 | - | 1655.7 | - | - | - | - | - | - | - | - | - | - | - | 0.17 |
| 1,8-menthadien-4-ol | 1183 | 1680 | 1189 | 1681 | 0.17 | 0.46 | 0.28 | 0.46 | 0.28 | 0.38 | 0.13 | 0.32 | - | - | - | - |
| Cryptone | - | 1674 | - | 1674.8 | - | 0.13 | - | - | - | - | - | - | - | - | - | - |
| Terpinen-4-ol | 1186 | 1601 | 1177 | 1601.2 | - | - | - | - | - | - | - | - | 2.24 | 2.39 | - | - |
| p-Cymen-8-ol | 1189 | - | 1183.9 | - | - | - | - | - | - | - | - | - | 0.09 | - | - | - |
| trans-p-Mentha-1(7),8-dien-2-ol | 1192 | 1789 | 1180.5 | 1791 | - | 0.27 | 0.26 | 0.25 | 0.23 | 0.23 | 0.23 | 0.27 | - | - | - | 0.18 |
| NI | 1192 | 1732 | - | - | 0.25 | 2.75 | - | - | - | - | - | - | - | - | - |  |
| α-Terpineol | 1199 | 1692 | 1189 | 1694 | - | - | - | - | - | - | - | - | 1.85 | 1.54 | 0.68 | 0.83 |
| NI | 1200 | - | - | - | - | - | - | - | - | - | 0.43 | - | - | - | - |  |
| octyl acetate | - | 1471 | - | 1474.6 | - | - | - | - | - | - | - | - | - | - | - | 0.11 |
| NI | 1201 | - | - | - | - | - | - | - | 0.56 | - | - | - | - | - | - | - |
| NI | 1201 | - | - | - | 0.44 | - | 0.56 | - | - | - | - | - | - | - | - | - |
| NI | 1204 | - | - | - | 0.65 | - | 0.84 | - | 0.85 | - | 0.86 | - | - | - | - | - |
| Decanal | 1205 | - | 1204 | - | - | - |  | - | - | - | - | - | 0.20 | - | 2.29 | - |
| NI | 1210 | - | - | - | - | - | 0.16 | - | - | - | 0.12 | - | - | - | - | - |
| trans-Carveol | 1222 | 1828 | 1217 | 1836.3 | 1.82 | 2.29 | 2.19 | 2.17 | 1.69 | 1.84 | 1.33 | 1.65 | 0.11 | 0.14 | 0.22 | 0.37 |
| Nerol | 1225 | 1791 | 1228.9 | 1794.6 | - | - | - | - | - | - | - | - | 0.21 | 0.18 | - | - |
| cis-p-Mentha-1(7),8-dien-2-ol | 1233 | 1879 | 1233 | 1894.9 | 0.14 | 0.16 | 0.17 | 0.16 | - | 0.13 | 0.16 | 0.20 | - | - | - | - |
| cis-Carveol | 1236 | 1858 | 1229 | 1854.4 | 0.76 | 1.05 | 0.96 | 1.00 | 0.87 | 0.85 | 0.56 | 0.81 | - | - | - | 0.21 |
| Neral | 1239 | 1676 | 1240 | 1678.5 | - | - | - | - | - | - | - | - | 1.90 | 1.77 | 0.41 | 0.50 |
| Carvone | 1249 | 1732 | 1243 | 1733.6 | 1.65 | 0.62 | 1.84 | 2.95 | 1.51 | 2.62 | 1.38 | 2.81 | - | 0.27 | 0.33 | 0.75 |
| Geraniol | 1249 | 1839 | 1255 | 1839.3 | - | 0.30 | - | 0.24 | - | 0.22 | - | 0.25 | 0.22 | 0.27 | - | 0.19 |
| NI | 1261 | - | - | - | - | - | - | - | - | - | - | - | 0.10 | - | - | - |
| Geranial | 1267 | 1724 | 1270 | 1725 | - | - | - | - | - | - | - | - | 1.69 | - | 0.29 | 0.55 |
| 1,5-Hexadiene, 2,5-dimethyl-3-methylene- | 1271 | - | - | - | - | - | 0.05 | - | - | - | - | - | - | - | - | - |
| NI | 1274 | 1746 | - | - | 0.11 | 0.43 | 0.14 | 0.40 | 0.19 | 0.30 | 0.13 | 0.26 | - | - | - | - |
| cis-Carvone oxide | 1279 | - | 1263 | - | - | - | 0.10 | - | - | - | - | - | - | - | - | - |
| Perilla aldehyde | 1281 | 1781 | 1273.4 | 1793.9 | 0.24 | 0.25 | 0.18 | 0.21 | 0.20 | 0.21 | 0.23 | 0.22 | - | - | 0.23 | 0.28 |
| NI | 1289 | 1947 | - | - | 0.45 | - | 0.51 | 0.47 | 0.37 | 0.43 | 0.47 | 0.42 | - | - | - |  |
| Limonen-10-ol | 1294 | 1985 | 1239 | 1979 | 0.15 | 0.17 | 0.22 | 0.16 | 0.16 | 0.20 | 0.17 | 0.19 | - | - | 0.23 | 0.32 |
| NI | - | 1979 | - | - | - | 0.08 | - | - | - | - | - | - | - | - | - | - |
| (R)-4-methyl-3-(3'-oxobutyl)pent-4-enal | - | 1947 | - | - | - | 0.55 | - | - | - | - | - | - | - | - | - | - |
| Perilla alcohol | 1303 | 1995 | 1296.3 | 2006.6 | 0.26 | 0.19 | 0.38 | 0.17 | 0.28 | 0.16 | 0.28 | 0.14 | - | - | - | - |
| Cyclohexene, 2-ethenyl-1,3,3-trimethyl- | 1308 | - | - | - | 1.48 | - | 1.51 | - | 1.28 | - | 1.45 | - | 0.15 | - | 0.62 | - |
| Limonene dioxide | 1312 | - | 1294 | - | - | - | 0.07 | - | - | - | - | - | - | - | - | - |
| (1S,4R)-p-Mentha-2,8-diene, 1-hydroperoxide | 1322 | - | - | - | - | - | - | - | - | - | - | - | - | - | 0.33 | - |
| NI | 1322 | - | - | - | 1.41 | - | 1.38 | - | 1.18 | - | 1.25 | - | 0.26 | - | - | - |
| NI | 1335 | - | - | - | 0.96 | - | 1.02 | - | 0.86 | - | 0.94 | - | - | - | - | - |
| NI | 1347 | - | - | - | - | - | - | - | - | - |  | - | 0.24 | - | - | - |
| Limonene diol | 1349 | - | 1321 | - | - | - | - | - | - | - |  | - | - | - | 0.27 | - |
| NI | 1349 | - | - | - | - | - | 0.17 | - | - | - | 0.33 | - | - | - | - | - |
| NI | 1355 | - | - | - | 0.25 | - | 0.33 | - | 0.36 | - | 0.40 | - | - | - | 0.32 | - |
| Neryl acetate | 1357 | 1716 | 1365 | 1718.1 | - | - | - | - | - | - |  | - | 2.57 | 1.98 | - | - |
| Decanoic acid | 1360 | - | 1375.5 | - | - | - | - | - | - | - |  | - | - | - | 0.35 | - |
| NI | 1361 | - | - | - | 1.52 | - | 1.47 | - | 1.12 | - | 1.31 | - | 0.19 | - | - | - |
| NI | 1367 | - | - | - | 0.27 | - | 0.26 | - | 0.24 | - | 0.21 | - | - | - | - | - |
| Geranyl acetate | 1376 | 1745 | 1381 | 1751 | - | - | - | - | - | - | - | - | 0.72 | 0.48 | - | - |
| NI | 1377 | - | - | - | 1.18 | - | 1.18 | - | 0.91 | - | 1.09 | - | - | - | 0.36 | - |
| α-Copaene | 1385 | 1498 | 1377 | 1491 | - | - | - | - | 0.14 | 0.30 | 0.10 | 0.34 | - | - | 0.45 | 2.59 |
| β-Bourbonene | 1394 | 1525 | 1384.2 | 1523.2 | - | - | - | - | - | - | - | - | 0.11 | - | - | 0.13 |
| β-cubebene | 1396 | 1542 | 1386.6 | 1541.7 | - | - | - | - | - | - | - | - | - | - | 0.59 | 0.23 |
| β-elemene | 1396 | 1592 | 1390.4 | 1590.9 | - | - | - | - | - | - | - | - | 0.11 | 0.10 | - | 0.15 |
| Dodecanal | 1409 | 1708 | 1407 | 1711.5 | - | - | - | - | - | - | 0.19 | - | - | - | 0.76 | 0.71 |
| cis-α-Bergamotene | 1420 | 1553 | 1414.5 | 1559.1 | - | 0.63 | - | 0.64 | - | 0.59 | - | 0.54 | 0.16 | 0.16 | - | 0.32 |
| β-Caryophyllene | 1433 | 1603 | 1428 | 1598.5 | - | - | - | - | - | - | - | - | 0.41 | - | 0.24 | 0.33 |
| trans-α-Bergamotene | 1440 | 1560 | 1434.5 | 1575.7 | - | 0.36 | - | 0.39 | - | 0.33 | - | 0.31 | 1.98 | - |  | 0.13 |
| β-Copaene | 1442 | 1599 | 1432 | 1579.8 | - | - | - | - | 0.10 | - | 0.11 | - | - | - | 0.52 | 0.48 |
| α-Himachalene | 1444 | - | 1445.1 | - | - | - | - | - | - | - | - | - | 0.08 | - | - |  |
| cis-β-Farnesene | 1454 | 1657 | 1445.9 | 1651.4 | - | - | - | - | - | - | - | - | 0.11 | 0.10 | - | 0.13 |
| β-Santalene | 1468 | - | 1462 | - | - | - | - | - | - | - | - | - | 0.16 | - | - | - |
| trans-β-Farnesene | 1492 | - | 1455.9 | - | - | - | - | - | - | - | - | - | 0.11 | - | - | - |
| β-Selinene | 1496 | - | 1485 | - | - | - | - | - | 0.11 | - | - | - | - | - | - | - |
| cis-α-Bisabolene | 1503 | - | 1503.1 | - | - | - | - | - | - | - | - | - | 0.36 | - | - | - |
| Velencene | 1504 | 1721 | 1491 | 1728.6 | - | - | - | - | 1.84 | 1.21 | 0.31 | 0.18 | - | - | 1.57 | 0.99 |
| β-Bisabolene | 1511 | 1723 | 1509 | 1727.5 | - | - | - | - | - | - | - | - | 3.07 | 3.60 | - | - |
| δ-Cadinene | 1526 | 1753 | 1523.2 | 1763.3 | - | - | - | - | - | - | - | - | - | - | 0.40 | 0.41 |
| α-7-Epi-selinene | 1534 | - | 1540 | - | - | - | - | - | 0.09 | - | - | - | - | - | - | - |
| Elemol | 1557 | - | 1549 | - | - | - | - | - | - | - | - | - | - | - | 0.26 | - |
| Caryophyllene oxide | 1597 | 1987 | 1583 | 1986.2 | - | - | - | - | 0.10 | - | - | - | 0.31 | 0.18 | 0.32 | - |
| NI | 1639 | - | - | - | - | - | - | - | - | - | - | - | 0.20 | - | - | - |
| α-Selin-11-en-4-ol | 1672 | - | 1654.9 | - | - | - | - | - | - | - | - | - | 0.21 | - | - | - |
| NI | 1683 | - | - | - | - | - | - | - | - | - | - | - | 0.12 | - | - | - |
| α-Bisabolol | 1694 | - | 1683 | - | - | - | - | - | - | - | - | - | 0.23 | - | - | - |
| β-Sinensal | 1697 | - | 1694 | - | - | - | - | - | - | - | - | - | - | - | 0.42 | - |
| Nootkatone | 1822 | - | 1823 | - | - | - | - | - | - | - | - | - | - | - | 0.35 | - |
| Hexadecanoic acid | 1955 | - | 1968.4 | - | - | - | - | - | - | - | - | - | - | - | 0.79 | - |
| Linoleic acid | 2127 | - | 2128.9 | - | - | - | - | - | - | - | - | - | - | - | 0.45 | - |
| trans-13-Octadecenoic acid | 2132 | - | - | - | - | - | - | - | - | - | - | - | - | - | 0.70 | - |
| Eicosane | 2259 | - | - | - | - | - | - | - | - | - | - | - | - | - | 0.18 | - |
| Tricosane | 2298 | - | 2300 | - | - | - | - | - | - | - | - | - | - | - | 0.27 | - |
| Docosane | 2373 | - | - | - | - | - | - | - | - | - | - | - | - | - | 0.15 | - |
| Bicyclo[3.3.0]oct-2-en-7-one, 6-methyl- | - | 1694 | - | - | - | 0.12 | - | 0.13 | - | 0.28 | - | 0.26 | - | - | - | - |
| NI | - | 1708 | - | - | - | - | - | - | - | - | - | 0.21 | - | - | - | - |
| NI | - | 1571 | - | - | - | - | - | - | - | - | - | - | - | 0.17 | - | - |
| NI | - | 1587 | - | - | - | - | - | - | - | - | - | - | - | 1.44 | - | - |
| TOTAL |  |  |  |  | 100.00 | 100.00 | 100.00 | 100.00 | 100.00 | 100.00 | 100.00 | 100.00 | 100.00 | 100.00 | 100.00 | 100.00 |

*OOPE = Orange oil phase essence, OPO = Orange peel oil, BOT = Brazilian orange terpenes, TLOP = Tahiti lime oil phase, OPOFF = Orange peel oil five fold and CT = Citrus terpenes.

^1^ Identification by GC/MS using non-polar and polar columns

^2^ LRI_C_: Linear retention index (Calculated)

^3^ LRI_L_: Linear retention index (Literature)

^4^ NP: non-polar column DB-5MS

^5^ P: polar column DB-WAX

^6^ Relative amounts of the identified compounds based on the area of each peak in the chromatogram.

NI: Not identified compound
